# Therapeutic Potential of Dietary Phenolic Acids

**DOI:** 10.1155/2015/823539

**Published:** 2015-09-09

**Authors:** Venkata Saibabu, Zeeshan Fatima, Luqman Ahmad Khan, Saif Hameed

**Affiliations:** ^1^Amity Institute of Biotechnology, Amity University Haryana, Gurgaon, Manesar 122413, India; ^2^Department of Biosciences, Jamia Millia Islamia, New Delhi 110025, India

## Abstract

Although modern lifestyle has eased the quality of human life, this lifestyle's related patterns have imparted negative effects on health to acquire multiple diseases. Many synthetic drugs are invented during the last millennium but most if not all of them possess several side effects and proved to be costly. Convincing evidences have established the premise that the phytotherapeutic potential of natural compounds and need of search for novel drugs from natural sources are of high priority. Phenolic acids (PAs) are a class of secondary metabolites spread throughout the plant kingdom and generally involved in plethora of cellular processes involved in plant growth and reproduction and also produced as defense mechanism to sustain various environmental stresses. Extensive research on PAs strongly suggests that consumption of these compounds hold promise to offer protection against various ailments in humans. This paper focuses on the naturally derived PAs and summarizes the action mechanisms of these compounds during disease conditions. Based on the available information in the literature, it is suggested that use of PAs as drugs is very promising; however more research and clinical trials are necessary before these bioactive molecules can be made for treatment. Finally this review provides greater awareness of the promise that natural PAs hold for use in the disease prevention and therapy.

## 1. Introduction

**Figure 1:**
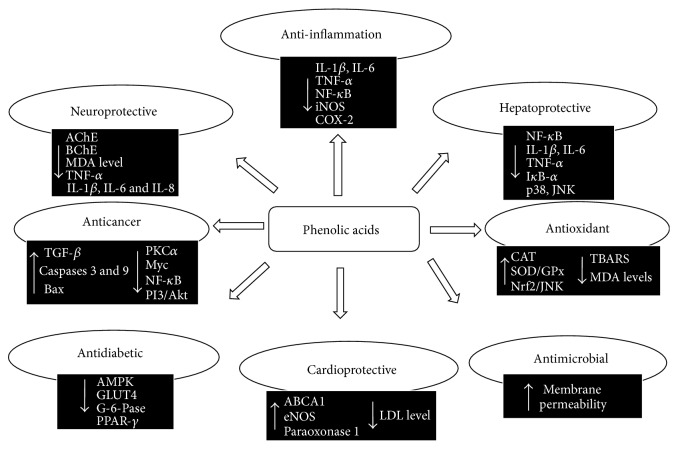
Key factors involved in health beneficial effects of natural PAs.

Since ancient times, plants and their products have been used as folk medicine in different parts of the world. It is believed that over 400,000 tropical flowering plant species have healing properties [1]. Several studies on medicinal plants determined the variety of compounds contributing to treatment of life-threatening diseases such as diabetes, cancer, and infections. Numbers of methods have been introduced for qualitative and quantitative analysis of plant secondary metabolites such as polyphenols, alkaloids, saponins, glycosides, resins, oleoresins, sesquiterpene, and lactones. These secondary compounds are involved in plant growth, development, reproduction, and disease resistance. Phenolic acids (PAs) are among the most common bioactive compounds throughout plant kingdom which have role in growth, reproduction, and defense against environmental stress and microorganisms [2, 3]. The antioxidant activities of PAs are extensively implemented in food industry as preservatives since antiquity. They also have function in plethora of important biological activities such as antiageing, reducing the risk of life-threatening diseases such as HIV, diabetes, CVDs, and cancer. Bulk information available about potential health benefits regarding PAs has led to an increased interest in PAs and this review illustrates the role that PAs possess in important biological conditions (Figure 1). A comprehensive literature search was conducted in PubMed and Google Scholar by using the following mesh keywords: (1) phenolic acids, (2) antioxidants, (3) anticancer, (4) cardioprotective, (5) antiulcer, (6) anti-diabetic, (7) antimicrobial, and (8) hepatoprotective. Thus, the aim of the present paper is to systematically review and summarize the literature supporting the health benefits of PAs at a common platform.

## 2. Phenolic Acids: Classification, Occurrence, and Bioavailability

PAs are aromatic carboxylic acids naturally appearing in the plant kingdom. The term phenolic acid describes a phenol ring that possesses at least one carboxylic acid functionality. However, in case of plant metabolites, these refer to a distinct group of organic acids which contains two distinguishing carbon frameworks, hydroxybenzoic and hydroxycinnamic acid structures. PAs are usually subclassified into benzoic acids containing seven carbon atoms (C6-C1) and cinnamic acids with nine carbon atoms (C6-C3).

**Figure 2:**
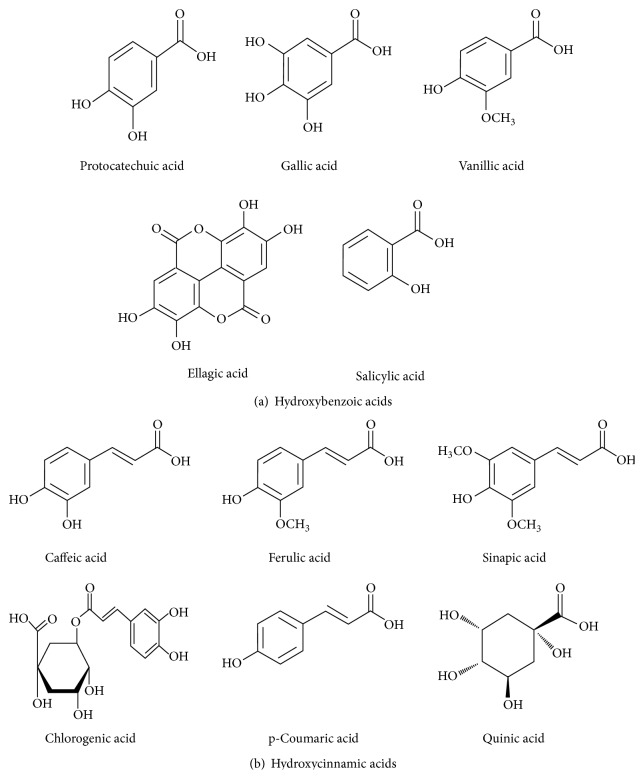
Structural classification of natural PAs discussed in the review.

PAs are widely distributed in the plant kingdom and found in a wide variety of nuts and fruits, such as raspberries, grapes, strawberries, walnuts, cranberries, and black currants. These are secondary metabolites derived from phenylalanine and tyrosine via shikimate/chorismate pathway. However, these compounds exist predominantly as hydroxybenzoic acids which include gallic acid (GA), salicylic acid (SA), protocatechuic acid (PCA), ellagic acid (EA), and gentisic acid (GeA) and hydroxycinnamic acids which include p-coumaric acid (p-CA), caffeic acid (CA), ferulic acid (FA), chlorogenic acid (CGA), and sinapic acids (SA) or alternatively may occur as conjugated forms (Figure 2).

The chemical properties of PAs in terms of availability of the phenolic hydroxyl groups predict their activity* in vitro*. The effective* in vivo* potential of PAs depends on multiple factors such as quantity of the compound ingested, absorbed, and/or metabolized, plasma and/or tissue concentrations, type and amount of single PA, and synergistic effects. However, the chemical structure of phenolic compounds differently affects all the factors described above. For instance, sulfate derivatives of FA and CA showed lower activities when compared with the antioxidant activities of the parental phenolic acids indicating the importance of the phenolic acids and their metabolites [4].

## 3. Antioxidant Properties

Biological system generates reactive oxygen species (ROS) as the byproduct of various metabolic activities and mitochondria are said to be major site responsible for ROS production. These free radicals are highly unstable or reactive and can cause direct damage to cell components such as nucleic acids, lipids, and proteins [5]. ROS is the major contributor to many diseases which include CVDs, cancer, age-related decline in the immune system, and degenerative diseases such as Parkinson's and Alzheimer's disease. Human body possesses several stress response enzymes including superoxide dismutase (SOD) and catalase (CAT) to counteract radical-induced damage. PAs counteract both ROS and RNS-induced cell damage by their direct free radical scavenging activity as well as the upregulation of the HO/BVR system, superoxide dismutase (SOD), and catalase (CAT) whose final aim is to detoxify ROS and RNS.

Oxidative stress has been implicated in the pathogenesis of multiple cardiovascular complications. Antioxidant role of FA in cardioprotection is well studied [6]. In hypersensitive rats, treatment with FA for 4–12 weeks increased SOD and CAT activities. FA treatment also increased SOD and CAT levels in myocardium and pancreatic tissue of streptozotocin-induced diabetic rats in dose and time dependent manner [7].

Antioxidant activities of PCA along with known antioxidant Trolox were measured* in vitro* using various antioxidant assays including 1,1-diphenyl-2-picrylhydrazyl (DPPH^•^), 2,2′-azino-bis(3-ethylbenzthiazoline-6-sulfonic acid) (ABTS^+•^), superoxide anion radicals (O_2_^−•^) and hydroxyl radical (^•^OH) scavenging activity, ferric ions (Fe^3+^) and cupric ions (Cu^2+^) reducing power, ferrous ions (Fe^2+^), and cupric ions (Cu^2+^) chelating activity. The antioxidant activity of PCA could be attributed to both its transition metal ions chelation and by free radicals scavenging via donating hydrogen atom (H^•^) or electron [8].

Oral administration of vanillic acid (VA) at high doses (100 mg/kg) increased the antioxidant status by reducing lipid peroxidation and increased the SOD, CAT, and glutathione peroxidase (GPx) activities and reduced glutathione (GSH) levels in cisplatin induced nephrotoxicity in albino rats [9]. Similarly, GA consumption diminished this harmful effect via its antioxidant action by inhibiting the initiation and/or propagation of the lipid peroxidation [10]. In the later studies same group investigated the antioxidant properties of GA derived from* Peltiphyllum peltatum* against sodium fluoride (NaF-) induced hepatotoxicity in rats [11]. Pretreatment of animals with GA could mitigate the NaF-induced hepatotoxicity through suppression of lipid peroxidation expressed in reduced Thiobarbituric Acid Reactive Substances (TBARS) levels, restoration of GPx levels, CAT, and SOD corroborating its antioxidative role.

Antioxidant properties of PAs depend on not only their direct activity as a scavenger but also their capacity to strengthen the endogenous antioxidant defenses. Nuclear factor erythroid 2- (NFE2-) related factor 2 (Nrf2) is a key transcription factor which strictly regulates the antioxidant/detoxifying enzyme genes by antioxidant response elements, present in the promoter region of those genes [12]. PAs such as PCA have been demonstrated to induce the ARE-dependent antioxidant/detoxifying phase II enzymes such as GR and GPx by activating the transcription factor Nrf2 through JNK-mediated phosphorylation [13]. Similarly, PCA induces antiapoptotic mechanism that likely related to the activation of the JNK-mediated survival signals that strengthen the cellular antioxidant defenses rather than antioxidant power of PCA. PAs induce phase II hepatic antioxidant enzyme and increase the antioxidant status of liver [14]. PAs including GEA, GA, p-CA, and FA selectively induce hepatic mRNA transcripts for CuZnSOD, GPx, and catalase likely through upregulation of gene transcription as well as the Nrf2 transcription factor indicating their potential antioxidant role in liver [15].

## 4. Antiulcer Activity

Gastric ulcer is a recurrent chronic illness caused by both endogenous and exogenous factors which include pepsin, stress, and noxious factors such as nonsteroidal anti-inflammatory drugs,* Helicobacter pylori* bacteria, smoking, and alcohol consumption [16]. Natural PAs have been investigated for their antiulcer activities which are apparent from wide ranges of studies in experimental animals. GA has been reported to be antiulcer in aspirin plus pylorus ligation model. GA exerts this effect by improving mucosal defensive with activation of antioxidant parameters and inhibition of some toxic oxidant parameters. Similarly, GA in combination with famotidine, an antiulcer drug, synergistically protected the experimentally induced peptic ulcer models [17]. Animals treated with low dose combinations like GA (50 mg/kg) + FM (10 mg/kg) along with high dose GA (100 mg/kg) + FM (10 mg/kg) showed significant ulcer protective effect induced by aspirin plus pylorus ligation.

Gastroprotective properties of another phenolic acid, EA, have also been reported. EA are well documented in acute and chronic ulcer rat models [18]. The gastroprotective properties of EA are partly due to increased endogenous production of nitric oxide which is an antioxidant effect by replenishing non-protein-sulfhydryls and attenuation of TNF-*α*, whereas in indomethacin ulcer, gastroprotective properties are partly due to attenuation of elevated levels of TNF-*α*, interferon-*γ*, and interleukins 4 and 6.

## 5. Antidiabetic Activity

Impairment in glucose metabolism leads to physiological imbalance with the onset of the hyperglycemia and subsequently diabetes mellitus which can be either type 1 or type 2. It is well known that several physiological parameters of the body get altered in the diabetic conditions. Long term effects of diabetes may cause development of specific complications such as retinopathy, nephropathy, neuropathy, foot ulcers, sexual dysfunctions, and cardiovascular complications.

Insulin stimulates glucose transport by inducing the translocation of glucose transporter 4 (GLUT4) to the plasma membrane which is mediated by two major pathways, an insulin independent AMPK pathway and an insulin dependent PI3K pathway. The antidiabetic effects of natural PAs are well documented as evident from wide range of studies. CGA could enhance the glucose uptake in L6 myotubes via increasing expression of GLUT4 and PPAR-*γ* transcript [19]. Similarly, PCA also exerts its insulin-like activity in human omental adipocytes. PCA induces the glucose uptake associated with enhanced GLUT4 translocation and increased PPARg transcript [20]. GA derived from* sea buckthorn* leaf extract stimulated GLUT4 translocation and glucose transport in a concentration dependent manner with maximum stimulation at 10 *μ*M concentration [21]. Further, the role of atypical protein kinase Cf/k is revealed in GA mediated GLUT4 translocation and glucose uptake.

Similarly, CGA stimulates glucose transport in both skeletal muscle isolated from mice and L6 skeletal muscle cells [22]. Furthermore, in L6 myotubes, CGA increased glucose transport via GLUT4 transporter and in skeletal muscle via the activation of AMPK. CGA lowered AUC glucose, inhibited hepatic glucose-6-phosphatase (G-6-Pase) expression, and improved skeletal muscle glucose uptake and lipid profiles, in Lepr^db/db^ mice. Mechanistically, CGA activated AMPK leading to suppression of hepatic glucose production and fatty acid biosynthesis. Inhibition and knockdown of AMPK abrogating these metabolic alterations suggested that CGA can improve glucose and lipid metabolism via the activation of AMPK. Another phenolic acid p-CA modulated glucose and lipid metabolism via AMPK activation in L6 skeletal muscle cells [23]. p-CA also promoted fatty acid *β*-oxidation, decreased oleic acid-induced triglyceride accumulation, and enhanced glucose uptake proving its beneficial effects in improving or treating metabolic disorders.

## 6. Cardioprotective Activity

Cardiac disease is the major cause of morbidity and mortality among the noncommunicable diseases. Implication of cardiovascular complications including cardiomyopathy in diabetes can be attributed to hyperlipidemia, hyperglycemia, and oxidative stress which promote atherosclerosis [24–26]. Cardiovascular complications are higher in diabetic patients than nondiabetics [27]. Cardioprotective role of the two phenolic acids caffeic acid (CA) and ellagic acid (EA) has been reported in STZ-induced diabetic mice. Treatment with CA or EA markedly elevated the insulin secretion, which might attenuate the dyslipidemia, improved glycemic control, and diminished cardiac oxidative stress [28].

Atherosclerosis is a chronic inflammatory disease characterized by accumulation of leukocytes in the vascular wall. Platelets play essential role in formation of atherosclerotic plaques and thrombosis by coaggregating with leukocytes via P-selectin glycoprotein ligand-1 (PSGL-1) and P-selectin interactions [29, 30]. GA has been found to possess antiatherosclerotic activity by inhibiting platelet activation and its association with leukocytes, P-selectin expression stimulated by ADP which is likely to be through decreasing intracellular Ca^2+^ mobilization via regulating the signals of PKC*α*/p38MAPK and Akt/GSK3*β* [31]. Protocatechuic aldehyde (structurally similar to PCA) isolated from root of* Salvia miltiorrhiza* attenuated PDGF-induced proliferation and migration of VSMCs via (PI3K)/Akt and MAPK kinase pathways which regulate key enzymes associated with migration and proliferation of VSMCs [32]. It is reported that FA effectively reduced copper ion induced LDL oxidation and facilitated the uptake and degradation of cholesterol in the liver. p-CA is known to protect the LDL from ROS by scavenging ^•^OH* in vivo* and thereby reduce lipid peroxidation and serum LDL cholesterol levels [33].

Cardioprotective activities of EA have also been investigated. EA effectively reduces oxidative stress, lowers the levels of plasma lipids, and inhibits lipid peroxidation. EA reduced the uptake of oxidized LDL in murine macrophages by downregulating membrane expression of SR-B1 [34]. SR-B1 is a membrane receptor on macrophages responsible for the internalization of oxidized LDL that promotes cellular accumulation of cholesterol [35]. Similarly, EA also promotes cholesterol efflux in lipid-loaded macrophages by inducing membrane receptor ABCA1 expression. ABCA1 is a membrane transporter abundant in macrophages and plays a crucial role in cholesterol homeostasis, thereby protecting against atherosclerosis [36]. Salicylic acid (SA) treatment has been reported to prevent complications of atherosclerotic cardiovascular disease such as myocardial infarction and occlusive stroke [37]. These effects of SA are attributed to its platelet-inhibitory function and stimulation of Paraoxonase 1, an enzyme that protects the serum lipids from oxidation and can reduce macrophage foam cell formation and attenuates atherosclerosis development [38].

Hypertension is the most common cardiovascular disorder mainly affected by lifestyle and dietary habits [39]. Nitric oxide (NO) plays a key role in the physiologic control of blood pressure and myocardial injury. Alterations in NO synthesis or bioavailability can cause vasoconstriction and might be involved in the pathogenesis of hypertension. VA has been proven to act against cardiovascular complications aroused due to hypertension [40]. VA normalized hypertension and left ventricular function in N*ω*-nitro-L-arginine methyl ester (L-NAME) induced hypertensive rat models. L-NAME is NO synthase inhibitor which causes reduction in its activity, leading to hypertension and arteriosclerosis [41–43]. Vanillic acid (VA) possessed cardioprotective effect, which is evident by lowered cardiac marker enzymes (CK, CK-MB, and LDH), left ventricular functions, improved tissue nitric oxide metabolite levels, and upregulated mRNA expression of eNOS in L-NAME induced hypertensive rat.

## 7. Anticancer Activity

Cancer is one of the leading causes of the deaths worldwide and chemotherapy is mainly used to treat cancer. However, occurrence of severe side effects of the drugs led to search for alternatives. Phytochemicals exert their anticancer activities by protecting critical cellular components (DNA, proteins, and lipids) from oxidative insult and interfere with proliferative activity and induction of apoptosis of cancer cells. Epidemiological and experimental studies confirmed that consumption of dietary products such as fruits and vegetables may have significant impact on the development of different types of cancers. EA is proven to be capable of decreasing oxidative stress, a hallmark of developing cancer. EA downregulates the expression and activity of PKC*α* in lymphoma bearing mice via decreasing the oxidative stress as evident by reduced lipid peroxidation and protein carbonylation [44]. Similarly, EA downregulates the expression of oncogene Myc and upregulates tumor suppressor gene TGF-*β* in lymphoma bearing mice. EA also exerts apoptosis through targeting PI3K/Akt kinases that in turn resulted in attenuation of its downstream Bcl-2 family proteins in 1,2-dimethyl hydrazine- (DMH-) induced rat colon carcinogenesis [45]. EA could stimulate the apoptosis by decreasing NF-*κ*B activity, thus activating the mitochondrial death pathway associated with caspase 3 activation and cytochrome C release [46]. Additionally EA also lowers the expression of cyclins against inflammation mediated cell proliferation by downregulation of NF-*κ*B in EA-administered rats. Induction of apoptosis features were observed in prostate cancer using the transgenic rat for adenocarcinoma of prostate (TRAP) model and human prostate cancer cell line (LNCaP). These findings showed that EA inhibits the early stage of prostate carcinogenesis through induction of apoptosis via activation of caspase 3 [47]. Similarly inactivation of phosphatidyl inositol 3-kinase (PI3K)/Akt pathway, Bcl-2 downregulation and increased expression of Bax, caspase 3, and cytochrome c, increased annexin V apoptotic cells, and DNA fragmentation were observed in human adenocarcinoma cells [46]. Cytotoxic effects of EA have been reported in TSGH8301 [48]. Cells treated with EA resulted in arresting cell cycle at G0/G1, promoted ROS and Ca^2+^ production, activities of caspases 9 and 3, and decreased the level of ΔΨ*m*. EA and Embelin isolated from* Ardisia japonica* at low concentrations synergistically increased the apoptosis thereby decreasing the proliferation* in vitro*. Similarly, EA alone or in combination with Embelin alone decreased tumor size and tumor cellularity in a subcutaneous xenograft mouse model of pancreatic cancer.

Angiogenesis is a critical process in tumor development and metastasis. PAs have been showed to inhibit the angiogenesis in various cancer cell lines and* in vivo*. Caffeic acid phenethyl ester (CAPE), a major medicinal component of propolis, showed dose dependent VEGF secretion in MDA-231 cells and formation of capillary-like tubes by endothelial cells, implicate on antiangiogenesis [49]. Antiangiogenic actions of p-CA have been studied in the ECV304 human endothelial cell line. p-CA reduced the mRNA levels of two important angiogenic factors VEGF and bFGF that stimulate permeability, proliferation, and tube formation of endothelial cells [50].

Tumor metastasis is a complex cascade that is accompanied by metalloproteinase (MMP) upregulation and extracellular matrix degradation. Metastasis allows cancer cells to proliferate and invade blood or lymphatic system, further enhancing cancer cell invasion and worsening prognosis. Pomegranate juice (*Punica granatum*) and its three constituents luteolin, ellagic acid, and punicic acid (L + EA + P) have been shown to interfere with multiple biological processes such as suppression of cell growth, increased cell adhesion, inhibition of cell migration, and inhibition of chemotaxis towards proteins (SDF1*α*) involved in breast cancer metastasis [51]. Similarly, L + EA + P exhibited beneficial effect on metastasis of prostate cancer in SCID mouse tumor model and *Pten* ^−/−^,* K-rasG* ^*12D*^ prostate tumors. Studies were conducted by injecting luciferase-expressing human PCa cells in the region of the prostate ectopically and monitored the tumor progression with bioluminescence imaging weekly. Results revealed that L + E + P inhibit PC-3M-luc primary tumor growth and suppress the CXCL12/CXCR4, implicated as therapeutic target for metastasis [52]. Further, L + E + P significantly inhibited the growth and metastasis* in vivo*. GA is known to inhibit metastasis and invasive growth of gastric cancer cells via increased expression of RhoB, downregulation of AKT/small GTPase signals, and inhibition of NF-*κ*B activity [53].

## 8. Anti-Inflammatory Activity

Inflammation is the complex biological process of vascular tissues to harmful stimuli, such as pathogens, chemical irritants, and damaged cells or tissue. It is a protective attempt by the organism in which immune system plays a major role. Inflammatory response includes (1) migration of immune cells from blood vessels and release of mediators at the site of damage followed by (2) recruitment of inflammatory cells, (3) release of ROS, RNS, and proinflammatory cytokines to eliminate foreign pathogens, and (4) repairing injured tissues. Though acute inflammation is rapid and self-limiting, prolonged inflammation causes various chronic disorders [54]. The link between chronic inflammation and various diseases such as cancer, metabolic disorder, type II diabetes, arthritis, autoimmune diseases, neurological diseases, pulmonary diseases, and cardiovascular complications is well known. Among other natural bioactive compounds, PAs are widely recognized for their anti-inflammatory properties in wide range disease conditions.

Platelets are key mediators of inflammation that may trigger an inflammatory response in vessel wall early in the development of atherosclerosis and contribute to the destabilization of advanced atherosclerotic lesions [55]. CGA possesses antiplatelet activity by reducing release of atherosclerotic-related inflammatory mediators (sP-selectin, IL-1*β*, sCD40L, and CCL5) and inhibits* in vivo* thrombus formation [56]. In addition, antiplatelet and antithrombotic effects shown by CGA are associated with A_2A_ receptor activation. Similarly, CGA induced an anti-inflammatory effect in lipopolysaccharide- (LPS-) inflamed murine RAW 264.7 macrophage cells through (1) suppression of proinflammatory cytokines such as IL-1*β*, TNF-*α*, and IL-6, as well as the chemokine CXCL1 through downregulation of NF-*κ*B; (2) decreased NO production mediated by downregulation of iNOS; and (3) inhibition of an important adhesion molecule Ninjurin 1 (Ninj 1) [57]. CA attenuated LPS-induced NO production possibly by inhibiting phosphorylation of p38MAPK and JNK1/2, suggesting that CA selectively inhibit different LPS-induced proinflammatory signaling cascades in RAW 264.7 macrophages [58]. Further, CGA showed antiosteoarthritis (anti-OA) properties in IL-1*β*-stimulated chondrocytes [54]. CGA suppressed the production of NO and PGE2 via downregulating the expression of iNOS and COX-2 which are implicated in the pathogenesis of OA [59]. In another study, CGA leads to attenuation in TLR4-mediated inflammatory responses such as NO and PGE2 production in LPS-treated RAW 264.7 cells and improves HCl/EtOH-induced acute gastritis symptoms [60].

## 9. Neuroprotective Activity

Neuroprotection means prevention of nerve cells from dying and usually involves an intervention of drug treatment. It is a mechanism used to protect neuronal injury or degeneration of CNS following acute disorders. The goal of neuroprotection is to limit neuronal dysfunction after injury and attempt to maintain the possible integrity of cellular interactions in the brain resulting in undisturbed neural function. There are wide ranges of natural compounds from plants and in particular PAs have been the focus of research. These compounds may act as free radical scavengers, antiexcitotoxic agents, apoptosis inhibitors, and so forth. Two hydroxycinnamic acids CGA and CA have been shown to be associated with anti-Alzheimer's properties [61]. Mechanistically, these properties could be attributed to inhibition of BChE and AChE activities; thus butyrylcholine breaks down and slows down acetylcholine* in vitro*. Furthermore, CGA and CA decrease the malondialdehyde (MDA) formed due to prooxidants such as FeSO_4_, sodium nitroprusside, and quinolinic acid. Recent studies demonstrated the novel evidence explaining the neurological effects of CGA. Voltage gated potassium channels (Kv) play crucial role in the electrophysiological processes of sensory neurons and have been identified as potential therapeutic targets for inflammation and neuropathic pain disorders. The effects of CGA on the two main subtypes of Kv in trigeminal ganglion (TG) neurons, namely, the IK,V and IK,A channels, are also highlighted [62]. Upon CGA treatment, activation and inactivation currents of both IK,V and IK,A were significantly shifted toward depolarization, which implies that Kv is triggered at a lower threshold with a prolonged duration thus enhancing Kv activities in both IK,A and IK,V channels. This would gradually decrease the excitability of neurons during trigeminal hyperalgesic conditions in neuropathic and inflammatory pain.

Functional restoration of injured spinal cord by self-assembled nanoparticles composed of FA modified glycol chitosan (FA-GC) has been investigated to a considerable extent [63]. These nanoparticles protected primary neurons from glutamate-induced excitotoxicity* in vitro*. In addition, significant recovery in locomotor function was observed in a spinal cord contusion injury rat model that was intravenously administered FA-GC nanoparticles at 2 h after injury. Further, histological analysis revealed that FA-GC treatment significantly preserved axons and myelin and also reduced cavity volume, inflammatory response, and astrogliosis at the lesion site.

The accumulation of A*β* deposit owing to the metabolic disorders in the brain is key step in the pathogenesis of Alzheimer's disease (AD). A*β* can stimulate chronic neuroinflammation response and worsen the neurological degradation. Two genes, namely, presenilins 1 and 2 (PS1 and PS2) genes, or the amyloid-protein precursor (APP) plays an important role in the processing of A*β*. PCA derived from* Radix Salviae Miltiorrhizae* has been found to have a protective effect on improving cognitive deficits in STZ-induced AD rats [64]. The Morris water maze test revealed that PCA (100 mg/kg) significantly prolonged the mean latency time and the path length of A*β*PP/PS1 mice. PCA reduced the number of A*β* positive expressions in the hippocampus and cerebral cortex of A*β*PP/PS1 mice by immunocytochemical assay with Congo red staining and decreased remarkably APP expression levels by western blot analysis. In addition, PCA improves the cognitive deficits of AD animal by decreasing the levels of inflammatory cytokines including TNF-*α*, IL-1*β*, IL-6, and IL-8.

## 10. Hepatoprotective Activity

Liver diseases are a major health problem which causes high mortality and morbidity affecting the human of all ages. Liver diseases are classified as autoimmune hepatitis, alcoholic liver disease, and viral hepatitis. Despite advances in modern medicine, still there is lack of effective drug that completely cures liver complications. Therefore, there is urgent need for search of effective drugs to replace current drugs. Natural PAs are undoubtedly valuable as a source of new medicinal agents. Hepatoprotective effect of EA in concanavalin A- (Con A-) induced hepatitis mice model has been evaluated [65]. Pretreatment with EA significantly decreased the expression levels of the toll-like receptor 4 (TLR4) and TLR2 implicated in hepatitis possibly by inflammation. Further, EA decreased the expression of inflammatory cytokines and in turn inhibit the phosphorylation of MAPK and nuclear translocation of NF-*κ*B. This may account for a reduction in TNF-*α*, IL-6, and IL-1*β* expression by inhibition of NF-*κ*B-mediated transcriptional activation and I*κ*B-*α* degradation and prevention of JNK, p38, and ERK MAPK phosphorylation.

Resistin is a hormone implicated in enhanced hepatic steatosis via downregulation of content and activities of mitochondria [38]. EA reduced serum resistin levels and improved hepatic steatosis and serum lipid profile in high-fat fed obese diabetic KK-Ay mice [66]. EA supplementation also improved hepatic steatosis by reducing triglycerides, which play key role in liver steatosis and ameliorated serum HDLC and non-HDLC levels which might be caused by reduced serum resistin levels. Moreover, EA upregulated mRNA levels of PPARa and its target genes including cpt1a suggesting that EA acts as transcriptional activator of PPARa in the liver.

## 11. Antiaging Activity

Aging is the accumulation of changes over time associated with increasing susceptibility to different diseases and ultimately leading to death. Several hypotheses have been proposed to explain how aging occurs. However, many evidences led to the general acceptance of the oxidative stress theory that the accumulation of molecular damage caused by reactive oxygen species is a major factor in aging. PCA derived from dried fruits of* Alpinia oxyphylla* Miq. showed strong ability to attenuate ageing alterations of antioxidative defense systems in spleen and liver male Sprague-Dawley rats [67]. Intraperitoneal injection of PCA showed effects on splenic weight, antioxidant enzyme activities, and MDA levels in spleen and liver of rats. PCA isolated from* Veronica peregrine* exhibited significant antiaging effects on* Caenorhabditis elegans* model system [68]. In the presence of PCA, lifespan of the wild-type worms increased in a dose dependent manner. PCA also improved tolerance of worms against heat shock and osmotic and oxidative stress which might result in extension of lifespan. Pharyngeal pumping rate and progeny production are the two factors evidenced for prolonged lifespan and improved longevity. Interestingly, both factors were significantly reduced after PCA exposure. In addition, PCA-treated aged worms showed improved body movement compared to untreated controls suggesting PCA could enhance health span as well as lifespan.

## 12. Antimicrobial Activity

Illnesses resulting from pathogens and the emergence of resistance to conventional antibiotics are vital concern in public health and demand new antimicrobial compounds [69, 70]. Plants do not possess immune system like animals but instead synthesize phenolic compounds in response to the presence of pathogens, herbivores, and insects [71]. Research on natural products has demonstrated significant progress in the discovery of novel compounds with antimicrobial activity [72–74].

The antibacterial activities of PAs have been demonstrated in various studies on different pathogens.* Veronica montana* L. water extract and its main phenolic constituent, PCA, showed being highly effective against the growth of* Listeria monocytogenes* which causes listeriosis, which occurs mostly in elderly people, immune-compromised patients, and pregnant women [75, 76]. The mechanism of action of PCA towards* L. monocytogenes* appears to be alteration of permeability of bacterial cytoplasmic membrane. Antibacterial activity and mode of action of two phenolic acids have been investigated on* E. coli*,* P. aeruginosa*,* S. aureus*, and* L. monocytogenes*. It was found that FA and GA had antimicrobial activity against the bacteria tested with MIC of 500 mg/mL for* P. aeruginosa*; 1500 mg/mL for* E. coli*; 1750 mg/mL for* S. aureus*; and 2000 mg/mL for* L. monocytogenes* with GA. Similarly, FA showed the antimicrobial activity with MIC of 100 mg/mL for* E. coli* and* P. aeruginosa* and 1100 mg/mL and 1250 mg/mL for* S. aureus* and* L. monocytogenes*, respectively. CGA has been shown to have potent antibacterial and antibiofilm properties against emerging nosocomial pathogen* S. maltophilia*. The minimum inhibitory concentration was shown to be 8 to 16 *μ*g mL^−1^ and dose-dependently reduced the adhesion of* S. maltophilia* to polystyrene plate. Influence of subinhibitory concentrations of CGA on virulence associated factors of enterotoxigenic* S. aureus* has been investigated [77]. At subinhibitory concentrations (1.25 mg/mL), CGA significantly inhibited the hemolysis and coagulase titter. Reduced binding to fibrinogen and decreased production of SEA (an enterotoxin) were observed at concentrations ranging from 1/16MIC to 1/2MIC. Finally, CA markedly inhibited the expression of sea, hla, and agr genes in* S. aureus* [78].

## 13. Conclusion

The new lifestyles adopted by people have considerably enhanced the risk of acquiring various diseases. Extensive research on natural PAs provides new insight into the comprehension of the cellular and molecular mechanisms responsible for the immense potential therapeutic activity of these compounds against a number of human diseases. However, evidence of such properties has been collected from cellular and animal models, while clinical studies are still lacking. It is worth mentioning that further studies and clinical trials are needed to fully establish the preventive and therapeutic effectiveness of PAs and also to prove their safety for human consumption. Moreover, natural PAs also have the potential to drive the flux of funds to the corporate sector for investment in drug development which eventually may influence the quality of life for many patients. Certainly more intricate molecular mechanisms need to be investigated to further enhance their widespread usage and pharmaceutical potential.
